# MRI scans of the lumbar spine in lower back pain—why are we missing the elephant in the room?

**DOI:** 10.1093/jscr/rjaf1120

**Published:** 2026-01-26

**Authors:** Mansha Bhiryani, George Ampat

**Affiliations:** Countess of Chester Hospital NHS Foundation Trust, Countess of Chester Health Park Liverpool Rd, Chester CH2 1UL, United Kingdom; Trauma and Orthopaedics, School of Medicine, University of Liverpool, Cedar House, Ashton Street, Liverpool L69 3GE, United Kingdom

**Keywords:** lower back pain, paraspinal muscles, multifidus, radiology, magnetic resonance imaging

## Abstract

Lower back pain (LBP) is a leading cause of disability worldwide and a major contributor to healthcare costs. Diagnosing non-specific mechanical LBP remains difficult and often relies on exclusion. Current magnetic resonance imaging (MRI) reports typically emphasize disc degeneration, an age-related and often asymptomatic finding, while overlooking more clinically relevant factors like the state of the muscle. Fat infiltration and muscle wasting seen on MRI scans are common in LBP patients and are potentially reversible with targeted exercise rehabilitation. In a recent case, MRI revealed significant fat infiltration and muscle atrophy, yet the report focused solely on disc changes, missing the elephant in the room: the correctable muscular dysfunction. Since exercise is the cornerstone of LBP management, ignoring modifiable muscle health while highlighting irreversible and non-pathological age-related changes limits targeted treatment guidance. Routine reporting of muscle condition could lead to more precise rehabilitation and improved patient outcomes.

## Introduction

Lower back pain (LBP) is a leading musculoskeletal complaint, contributing significantly to global healthcare costs and accounting for 120 million lost workdays annually in the UK [1]. It is categorized as acute, subacute, or chronic (pain lasting >3 months). [2]. In the absence of any specified pathology, it is referred to as non-specific chronic LBP [2].

Lumbopelvic stability is maintained through the interaction of the active subunits, consisting of muscles and tendons, the passive subunits, consisting of the vertebral bodies and ligaments, and the neural subunits interconnecting the above two and providing feedback to these structures to ensure stability [3].

Passive spinal structures contain stretch receptors that relay proprioceptive input. When muscle strength is inadequate, these signals drive increased muscle recruitment to maintain stability [3].

Physical inactivity can cause structural and functional changes to this system, contributing to pain and instability. A sedentary lifestyle is associated with fatty degeneration of paraspinal muscles, thereby contributing to disability. [4]

The paraspinal muscles comprise of the psoas muscles (PM) anteriorly and the multifidus muscles (MF) and the erector spinae muscles (ES) posteriorly [3]. The MF muscle is a unisegmental muscle, providing fine segmental stability and control. In contrast, polysegmental muscles like ES facilitate gross spinal movements. Both are essential, but when MF is weaker, compensatory activation of larger polysegmental muscles can lead to reduced segmental stability and abnormal vertebral motion, increasing the risk of dysfunction or injury [3].

## Case report

A 74-year-old lady presented with a 2-year history of LBP. There were no red-flag symptoms to this back pain. She was typically fit and worked remotely from a desk. On examination, she had an adequate erect posture and gait. There was tenderness to palpation of the lumbosacral junction, the left sacroiliac joint and left L5/S1 facet joint. Forward flexion of the spine was achievable to about 10 cm proximal to her ankles. There was no sensory, motor or deep tendon reflex deficit. There were no upper motor signs.

**Figure 1:**
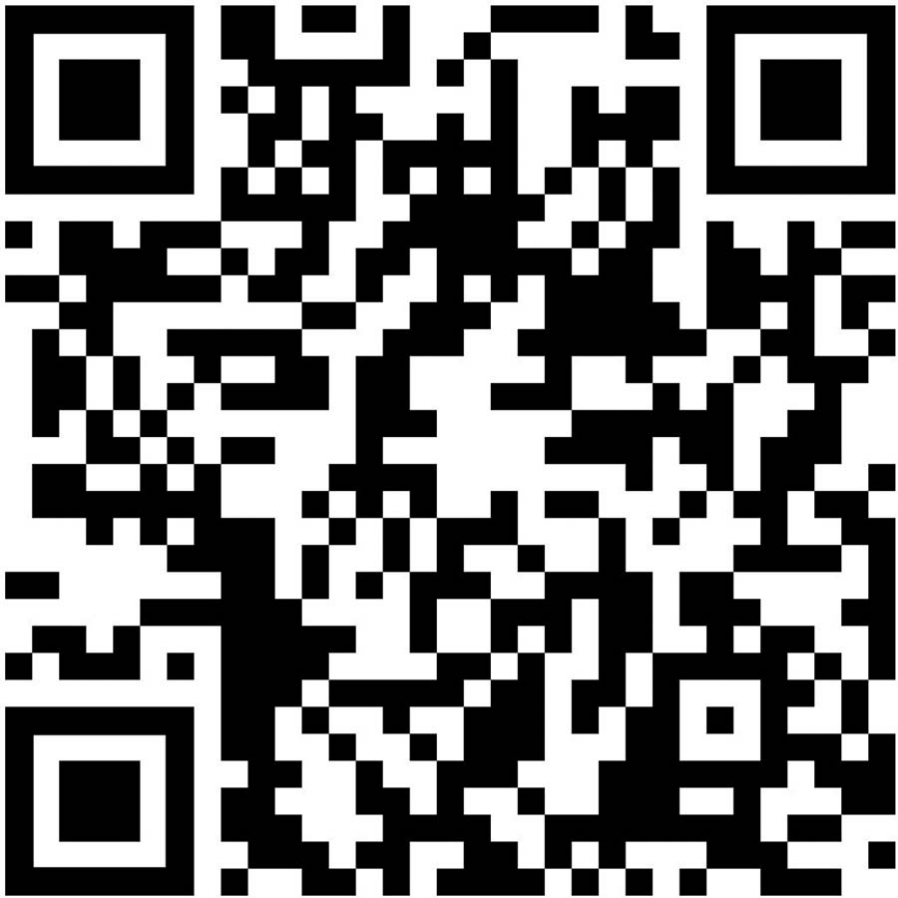
QR code providing the YouTube video by the senior author detailing the motor control exercises for lower back pain.

An magnetic resonance imaging (MRI) of the spine was requested due to the prolonged duration of symptoms and the patient's age. She was advised to perform motor control exercises for the LBP, and a YouTube link was provided (see Supplementary Material—Exercises for lower back pain, https://www.youtube.com/watch?v=xJiAqVsfpRc; QR code for Youtube link of motor control exercises by senior author, Fig. 1). The official MRI Spine scan report is reproduced in Table 1.

**Table 1:** The official MRI spine scan report provided by the radiology department

Noncontrast T1, T2, and STIR sagittal, T2 axial. Lower thoracic and lumbar vertebral bodies show normal height. The alignment is maintained. Vertebral body degenerative deformation at L2–L3–L4 with bridging osteophytes and discopathy bulging discs with subchondral erosions and associated endplate Modic type 2 changes. Bulging disc and posterior element degenerative hypertrophy causing mild spinal canal stenosis at L4/5 and bilateral synovitis. Degenerative changes at L5/S1 with Schmorl herniation and endplate Modic type 2 changes. The disc is bulged and touches both descending S1 nerve root. The disc at L3/4 is also bulged and touches both descending L4 nerve roots. No bone marrow oedema or vertebral bodies collapse. Visualized spinal cord and conus are unremarkable. Conclusion: No evidence of spondylolisthesis. High grade degenerative changes at L2, L3, and L4. Mild spinal canal stenosis at L4/5. At L5/S1 the disc touches both descending S1 nerve root and at L3/4 touches both descending L4 nerve roots.

Following the MRI scan, she continued performing the prescribed exercises and was more physically active. She was symptom-free at 6 months and satisfied with her progress. She was discharged from follow-up.

## Discussion

It is universally agreed that exercise is the principal treatment of LBP. In light of the association between exercise and muscle function, it is important to evaluate muscle strength and stability. MRI is a valuable and non-invasive tool that can help us assess muscle structure and integrity.

**Figure 2:**
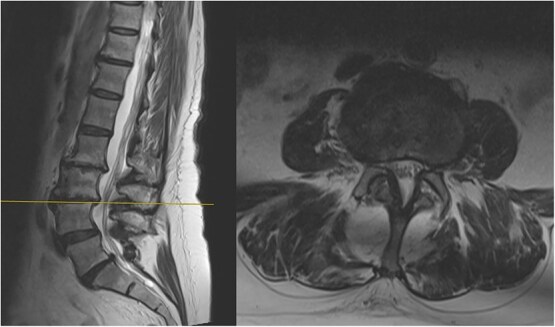
Sagittal and axial section at L3/4 showing no significant spinal stenosis or nerve root impingement.

**Figure 3:**
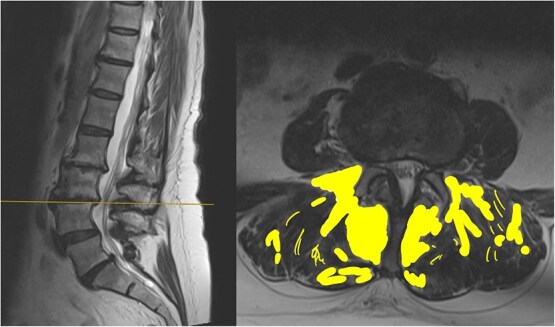
Sagittal and axial section at L3/4. The fat infiltration is highlighted in a yellow shade in this image.

The MRI report detailed the vertebral bodies, discs, and spinal cord but did not comment on the surrounding spinal musculature. On review of the images, no clear impingement of the spinal nerves was seen (Fig. 2). It was, however, noted that there was significant fat infiltration of the paraspinal muscles (Fig. 3).

The report had used the word degenerative on four occasions without acknowledging that this could be age-related. There was also no mention of the significant fat infiltration seen.

Fatty infiltration is a form of muscle sarcopenia. A sedentary job and lack of exercise can lead to weakening of the muscles, the changes of which can be eventually visualized on imaging. While spinal MRI is often discouraged unless clearly indicated, one advantage is its ability to detect fatty infiltration of muscles. Identifying such changes can reveal underlying muscular dysfunction, a potentially correctable problem, allowing for targeted rehabilitation.

As in this case, MRI reports commonly use terms such as ‘disc degeneration, bulge or narrowing’ which can have a significant psychological impact on patients, making them believe their condition is irreversible. This can lead to increased anxiety and reduced engagement in rehabilitation [5].

MRI scan reports should avoid potentially stigmatizing terms such as 'degenerative,' and instead emphasize modifiable factors like muscle weakness and fat infiltration [6] Reduced muscle size and fatty infiltration in lumbar musculature have been strongly linked to persistent LBP. MRI scans of LBP patients often show disc degeneration and atrophy of the PM, ES, and MF muscles, along with increased fat infiltration [7].

The effect of exercise on MF thickness remains debated. The FROST study found that high-intensity resistance training had minimal impact on MF fatty infiltration [7]. In contrast, Welch *et al*. [8] reported reduced fat infiltration and improvements in endurance, pain, and quality of life with resistance training. Despite some inconsistencies, resistance training is consistently associated with improved function and is an important element in LBP rehabilitation.

In line with this, our patient engaged in exercises targeting core activation, spinal stabilization, and lumbar endurance, supplemented with yoga and Pilates, disciplines grounded in similar principles. After several months, she reported notable improvements in both pain and function. This case, supported by existing literature, underscores the role of MF thickness and function in lumbopelvic stability and highlights the importance of addressing the MF in CLBP assessment and rehabilitation.

## References

[1] Du Bois M, Szpalski M, Donceel P. Patients at risk for long-term sick leave because of low back pain. Spine J 2009;9:350–9. 18790677 10.1016/j.spinee.2008.07.003

[2] Nicol V, Verdaguer C, Daste C, et al. Chronic low back pain: a narrative review of recent international guidelines for diagnosis and conservative treatment. JCM. 2023;12:1685. 36836220 10.3390/jcm12041685 PMC9964474

[3] Studnicka K, Ampat G. Lumbar stabilization. In: StatPearls [Internet]. Treasure Island (FL): StatPearls Publishing, 2025. Available from: http://www.ncbi.nlm.nih.gov/books/NBK562179/. 32965850

[4] Teichtahl AJ, Urquhart DM, Wang Y, et al. Physical inactivity is associated with narrower lumbar intervertebral discs, high fat content of paraspinal muscles and low back pain and disability. Arthritis Res Ther 2015;17:114. 25947906 10.1186/s13075-015-0629-y PMC4422596

[5] Myburgh C, Larsen TB, Kjaer P. ‘When the picture does not really tell the story’- a qualitative exploration of the MRI report of findings as a means for generating shared diagnostic meaning during the management of patients suffering from persistent spinal pain. Patient Educ Couns 2022;105:221–7. 34001396 10.1016/j.pec.2021.04.031

[6] Karran EL, Medalian Y, Hillier SL, et al. The impact of choosing words carefully: an online investigation into imaging reporting strategies and best practice care for low back pain. PeerJ. 2017;5:e4151. 29230375 10.7717/peerj.4151 PMC5723139

[7] Kircher K, Chaudry O, Nagel AM, et al. Effects of high-intensity training on fatty infiltration in paraspinal muscles in elderly males with osteosarcopenia – the randomized controlled FrOST study. BMC Geriatr 2024;24:141. 38326734 10.1186/s12877-024-04736-5 PMC10851592

[8] Welch N, Moran K, Antony J, et al. The effects of a free-weight-based resistance training intervention on pain, squat biomechanics and MRI-defined lumbar fat infiltration and functional cross-sectional area in those with chronic low back. BMJ Open Sport Exerc Med 2015;1:e000050. 10.1136/bmjsem-2015-000050 PMC5117021 27900136

